# Fabrication of Cu@Ag core-shell/nafion/polyalizarin: Applications to simultaneous electrocatalytic oxidation and reduction of nitrite in water samples

**DOI:** 10.1016/j.heliyon.2024.e40979

**Published:** 2024-12-07

**Authors:** Afshin Maleki, Nader Amini, Reza Rezaee, Mahdi Safari, Nader Marzban, Mehran seifi

**Affiliations:** a Environmental Health Research Center, Research Institute for Health Development, Kurdistan University of Medical Sciences, Sanandaj, Iran; b Leibniz Institute for Agricultural Engineering and Bioeconomy, Max-Eyth-Allee 100, 14469, Potsdam, Bornim, Germany

**Keywords:** Nitrite, Cu@Ag core-shell, Sensor, Conductive polymer, Simultaneously

## Abstract

In this study, a Cu@Ag core–shell was synthesized using a co-precipitation method. To create a new electrochemical sensor, a Cu@Ag core–shell with conductive polymers such as polyalizarin yellow R (PA) and Nafion (Nf) was immobilized on the surface of a glassy carbon electrode (Cu@Ag-Nf/PA/GCE). X-ray diffraction analysis (XRD), energy dispersive X-ray analysis (EDX), transmission electron microscopy (TEM), and Fourier Transform Infrared Spectroscopy (FTIR) techniques were employed to characterize the Cu@Ag-Nf/PA/GCE. This modified electrode was used to measure nitrite ions in the water samples. Electrochemical analysis of nitrite was conducted using differential pulse voltammetry (DPV) and cyclic voltammetry (CV) methods. For the first time, the results indicated that the Cu@Ag-Nf/PA nanocomposite demonstrated excellent performance in simultaneously electrocatalyzing oxidation at two specific potentials (0.17V and 0.98V denoted as OX1 and OX2 peaks) and one reduction potential (−0.42 V as a Red peak) for nitrite ions. This research showed various advantages, including applications in linear ranges, sensitivities, and detection limits in three potential areas (OX1, OX2, and Red) by elucidating the mechanism of action of the new electrode for detecting nitrite ions in water samples.

## Highlights

• A novel electrochemical sensor (Cu@Ag-Nf/PA/GCE) was Fabricated.
• The Cu@Ag-Nf/PA nanocompositeused for the simultaneous electrocatalytic oxidation and reductionof nitrite.
• This sensor was applied to analysis of nitrite water samples with satisfactory results.

## Graphical abstract

**Figure:**
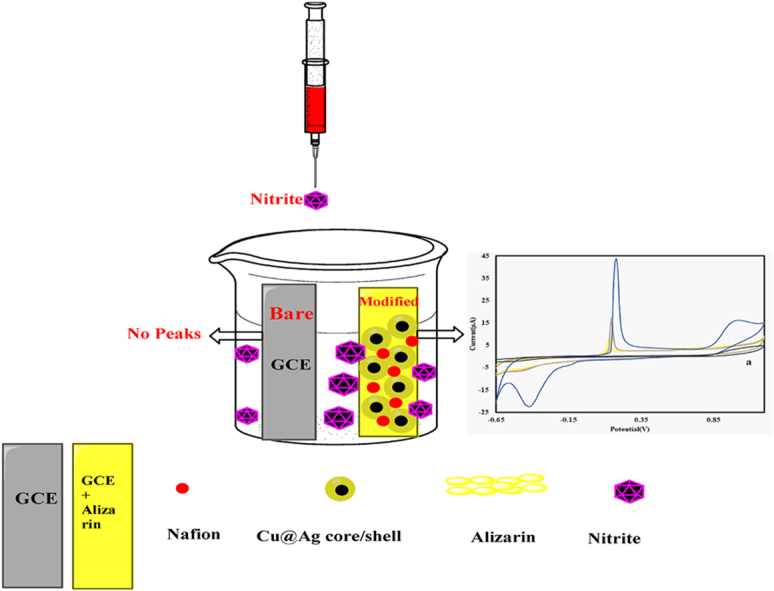

## 1 Introduction

**Scheme 1:**
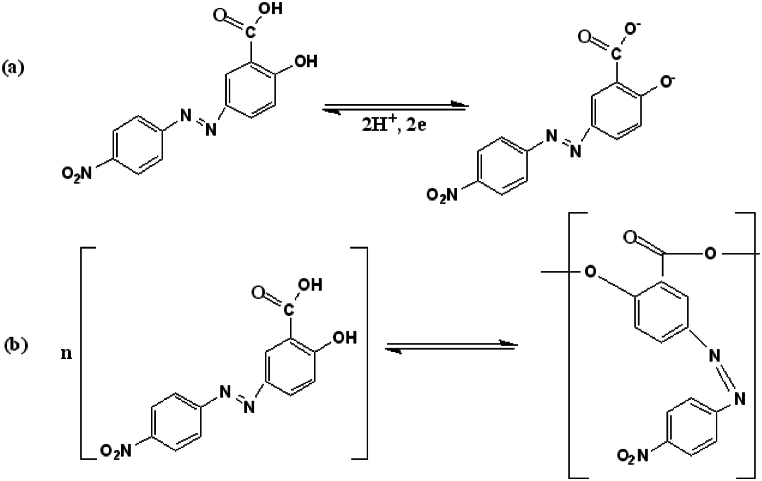
The steps involved in the electrochemical polymerization of alizarin. a Redox reaction of alizarin yellow R. b Formation of poly(alizarin) yellow R.

Nitrite (NO_2_^−^) is a reactive nitrogen species anion derived from a nitrogen oxoanion and is water-soluble. In humans, it acts as a metabolite and is essential for food and water safety [1]. Nitrite acts as a color fixative and antibacterial agent, improving the shelf life of processed milk, meats, cheese, and dyes, while functioning as a natural preservative [2]. It is commonly present in water from human activities and natural pollutants, mainly from chemical fertilizers and sewage [3,4]. Nitrite ions are highly toxic in large amounts, especially to humans and animals [5]. The International Agency for Research on Cancer has classified them as carcinogenic, and the World Health Organization (WHO) recommends a maximum permissible limit of 3 ppm (mg. L^−1^) for nitrites in drinking water [[6], [7], [8]]. Long-term exposure to nitrite ions can lead to serious health issues, including spontaneous abortion [9], gastric cancer [10], central nervous system disorders [11], and blue baby syndrome [12]. Detecting and quantifying nitrite concentrations in food and water is crucial [13,14]. Numerous scientific reports have outlined methods for measuring nitrite, including capillary electrophoresis, chemiluminescence, catalytic-spectrophotometry, spectrofluorometry, and chromatography [[15], [16], [17]]. Electrochemical sensors are advantageous due to their cost-effectiveness, rapid detection, sensitivity, ease of use, integration, and real-time analysis capabilities [18,19]. However, the electrochemical detection of nitrite on bare electrodes is slow, making electrode surface modification a viable solution. These modifications often involve materials such as metals [20,22], polymers [21], and core-shell nanomaterials [23] (see Scheme 1).

The size and shape of nanomaterials are essential in nanotechnology applications. Silver nanoparticles (AgNPs) are widely utilized in fields such as catalysis [24], electronics, and medical devices [[25], [26], [27], [28]], noted for their potential in electrochemical sensors, high electrical conductivity, and excellent oxidation stability [29]. However, AgNPs have limited electromigration resistance and high costs [30]. In contrast, copper nanoparticles (CuNPs) offer several advantages, including lower cost, strong redox properties, good catalytic activity, and high electrical conductivity [31,32], making them a common alternative to AgNPs. Nonetheless, CuNPs can oxidize to CuO or Cu_2_O under environmental conditions, which reduces their electrical conductivity and some applications [33,34]. Researchers have increasingly focused on bimetallic nanoparticles for their superior plasmonic, optical, biological, chemical, and catalytic properties compared to monometallic ones [35]. Copper/silver core-shell nanoparticles (Cu@Ag NPs) can mitigate the limitations of both CuNPs and AgNPs by enhancing the electromigration resistance of silver and the oxidation resistance of copper [36].

Conductive polymers are utilized for electrode modification owing to their numerous benefits, including reproducibility, strong adhesion to the electrode surface, stability, and homogeneity during chemical deposition. Conducting polymers, such as polypyrrole (PPy), Nafion (Nf), and polyalizarin (PA), are good choices for reducing ohmic drops in electroactive analytes. For instance, polyalizarin is an important conducting polymer used in electrochemistry. Combining nanomaterials, such as core-shell, with conductive polymers to create hybrid nanocomposites is also a fascinating approach in manufacturing electrochemical biosensors and sensors [[37], [38], [39], [40]].

In this work, a GCE, modified with a polyalizarin yellow R and Cu@Ag/Nafion (Cu@Ag/Nf/PA), is introduced for nitrite monitoring at two applied potentials (0.17 V and 0.98 V as OX1 and OX2 peaks) and one reduction potential (−0.42 V as Red peak). By combining the benefits of core-shell nanoparticles and conductive polymers, the resulting Cu@Ag/Nf/PA exhibited a low detection limit, simplicity, and excellent electrocatalytic activity for the oxidation and reduction of nitrite ions at three applied potentials. This sensor was used to analyze nitrite in water samples with good results.

## 2 Experimental

### 2.1 Reagents and materials

Ascorbic acid, Nafion, ethanol, alizarin yellow R, polyvinylpyrrolidone, NaNO_2_,CuSO_4_, HCl, NaOH, Na_2_HPO_4,_ AgNO_3_, NaH_2_PO_4_, KI, KIO_3_, Na_3_PO_4_, and alumina were purchased from Merck. The experiments were carried out at the laboratory temperature.

### 2.2 Instrumentation

A μ-Autolab electrochemical workstation (Eco Chemie U/techt, Netherlands) was utilized for electroanalysis. We employed a conventional three-electrode system consisting of a Pt wire as the auxiliary electrode, Ag/AgCl [KCl (sat)] as the reference electrode, and glassy carbon (unmodified or modified) as the working electrode. A pH meter was used to adjust the pH value.

### 2.3 Synthesis of Cu-Ag core-shell (Cu@Ag)

100 ml of deionized water in a 250 ml beaker was heated to 60 °C. Then, 2.0 g of polyvinylpyrrolidone (PVP) was added, and the mixture was stirred to achieve a clear solution with a 600 rpm for 60 min (Solution a). A 30 mL aliquot of the solution was transferred to a beaker, followed by the addition of 0.025 g of copper sulfate pentahydrate. This mixture was then subjected to continuous stirring for a duration of 10 min (Solution c). Concurrently, 100 mg of ascorbic acid, serving as a reducing agent, was dissolved in the remaining 70 mL of PVP solution. This process was conducted at a temperature of 60 °C, with stirring maintained for 60 min (Solution b). The solution (c) was gradually introduced to the solution b and agitated for 50 min at 60 °C; subsequently, the color of the solution transitioned to red, indicating the formation of CuNPs. The solution was then allowed to cool at room temperature for 1 h. 0.068 mg of AgNO_3_ was dissolved in 50 ml of water at 50 °C and and 500 rpm for 45 min (Solution d), followed by dropwise addition of ammonia solution (25 %) under continuous stirring (Solution e). Initially, a non-transparent solution was formed. Subsequently, upon the addition of the ammonia solution, the turbidity dissipated. The addition of ammonia was ceased when the solution became transparent. Additionally, 76 mg of Cu NPs was dispersed in 50 ml of deionized water and subsequently added to a solution at 50 °C, followed by continuous stirring for 90 min (Solution f). During the stirring process, the solution's color gradually transitioned to blue. The precipitate was cooled for 100 min, then the supernatant was removed and the Cu@Ag nanoparticles were collected. As-prepared sample of Cu@Ag core-shell was washed with deionized water and alcohol, centrifuged and dried in vacuum at 60 °C.

### 2.4 Electrode preparation and modification

Electrode preparation involved three steps. First, the bare glassy carbon electrode was polished with alumina (Al_2_O_3_) paste of varying grades to eliminate contaminants. Subsequently, it underwent ultrasonication in distilled water and EtOH for 5 min to further remove any contaminants [41].

**Fig. 1:**
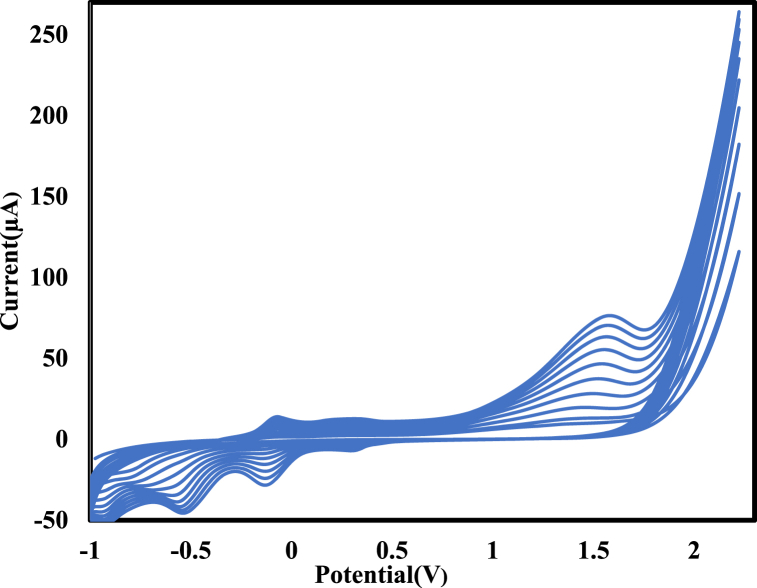
The successive cyclic voltammorams of the electropolymerization of PA on Cu@Ag-Nf electrode at potential scan rate 100 mVs^−1^.

In the second step, 5 μL (2 mg/ml) of Cu@Ag core-shell nanoparticles/Nafion (Nafion as a stabilizer agent of Cu@Ag on the bare GC working electrode) and EtOH solution were placed on the electrode surface and dried at 25 °C to fabricate the Cu@Ag-Nf electrode. To enhance the conductivity of the Cu@Ag-Nf electrode, the third step involved electropolymerization of alizarin yellow R at pH 7 and a potential range of −1 to 2.2 V. This was achieved using cyclic voltammetry with continuous scanning at a rate of 100 mV/s on a GCE to create the Cu@Ag-Nf/PA/GCE structure (Fig. 1).

The mechanism of electropolymerization of PA on the electrode surface is suggested as follows [42,43](Scheme 1).

## 3 Results and discussion

### 3.1 Morphological and characterization of Cu@Ag-Nf/PA/GCE and Cu@Ag core-shell

**Fig. 2:**
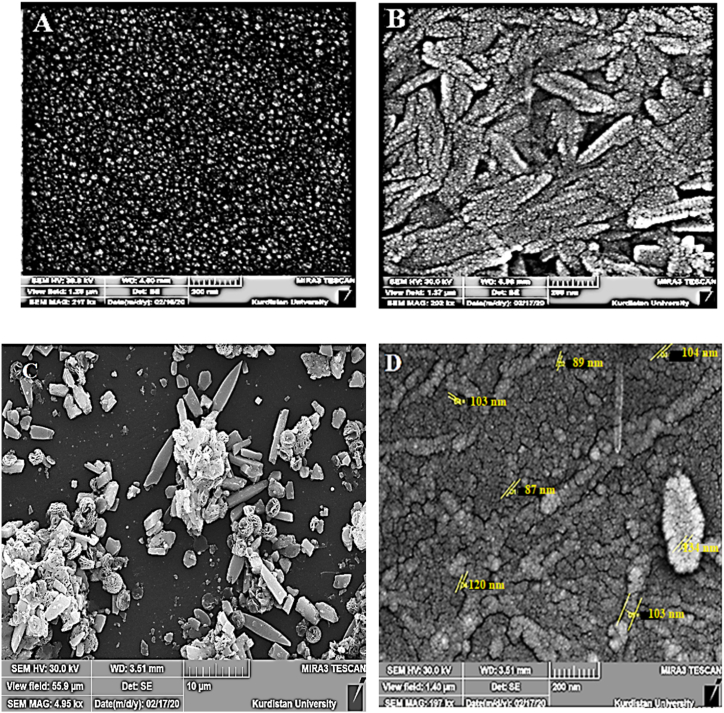
The SEM images for (A) bare, (B) Cu@Ag-Nf(C) and Cu@Ag-Nf/PA (D) glassy carbon electrodes.

SEM images of (A) bare, (B) Cu@Ag-Nf(C), and (D) Cu@Ag-Nf/PA glassy carbon electrodes are shown in Fig. 2. The surface images revealed distinct differences. Cu@Ag-Nf/PA was effectively deposited on the GC surface with particle sizes ranging from 10 to 89 nm.

**Fig. 3:**
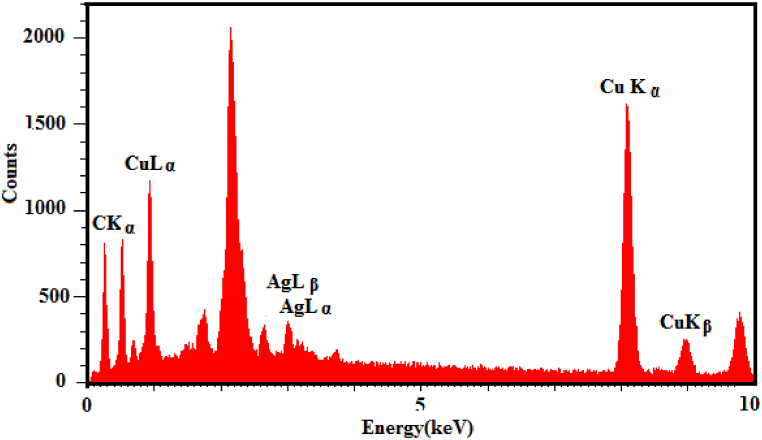
The EDX spectrum analysis was studied for Cu@Ag-Nf/PA/GCE.

EDX spectrum analysis was performed for Cu@Ag-Nf/PA/GCE (Fig. 3). As shown in Fig. 3, the peaks of Ag, Cu, C, and F were very strong. The high fluoride and carbon amounts were attributed to the Nafion and polyalizarin yellow R components, respectively.

**Fig. 4:**
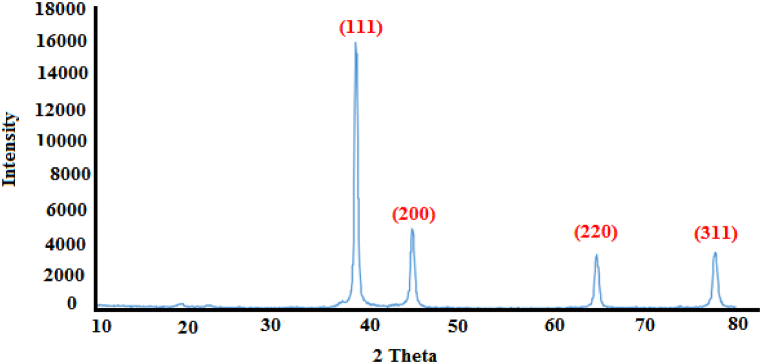
The XRD spectrum of Cu@Ag core-shell.

Fig. 4 shows the XRD spectrum of Cu@Ag core-shell. The obtained diffraction pattern is consistent with the patterns obtained in previous studies. It is exciting that the characteristic peaks from the Cu@Ag core-shell of Ag (111) are sharper than those of copper, which is due to the formation of a silver shell [44]. The low first peak at 2θ = 36.9° corresponds to the diffraction peak of CuO, which was assigned to the formation of copper oxides during catalyst preparation. The four peaks on the spectra of Cu@Ag at 2θ = 38.4°, 2θ = 44.6°, 2θ = 64.8° and 2θ = 77.8° corresponded to Ag (111), Ag (200), Ag (200) and Ag (311) planes. No diffraction peaks of Cu were observed for Cu@Ag, indicating that Cu metal and Ag metal are alloyed well [45].

**Fig. 5:**
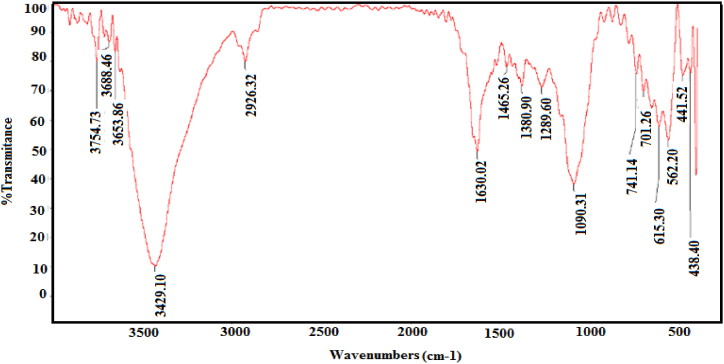
The Fourier transform infrared (FTIR) spectrum of Cu@Ag core-shell.

Analysis of the Fourier-transform infrared (FT-IR) spectral curve (Fig. 5) revealed characteristic absorption bands at 3440 cm^−1^ and 1636 cm^−1^, indicative of hydroxyl and carbonyl groups, respectively. The hydroxyl functionality may be attributed to either dehydroascorbic acid byproducts or moisture absorbed from the surrounding environment. The carbonyl signal is likely derived from both dehydroascorbic acid and polyvinyl pyrrolidone (PVP) molecules. Furthermore, a minor absorption peak at 1369 cm^−1^, associated with C-N stretching vibrations, provides strong evidence for the presence of a PVP capping layer. These spectral observations confirm the successful coating of Cu@Ag nanoparticles with dehydroascorbic acid and PVP. This capping agent layer plays a crucial role in preventing particle aggregation and enhancing resistance to oxidation [46].

### 3.2 Electrochemical detection of nitrite on Cu@Ag-Nf/PA/GC electrode

Cyclic voltammetry (CV) was used to perform electrochemical analysis of nitrite (NO_2_^−^) for the unmodified and modified electrodes. As shown inFig. 6, in the presence of NO2 − at the surface of the bare GC electrodes (a), no noticeable oxidation and reduction peaks were observed. However, at the surface of the Cu@Ag-Nf/PA/GCE, a silver oxidation peak appeared in the absence of nitrite (b). At the surface of the PA/GCE(c) and Cu@Ag-Nf/GCE (d), the oxidation and reduction peaks increased in the presence of nitrite (c). Finally, at the surface of Cu@Ag-Nf/PA/GCE (e) and in the presence of nitrite, two oxidation peaks (OX1 = 0.17 V OX2 = 0.98 V) and a reduction peak (Red = −0.42 V) were observed.

**Fig. 6:**
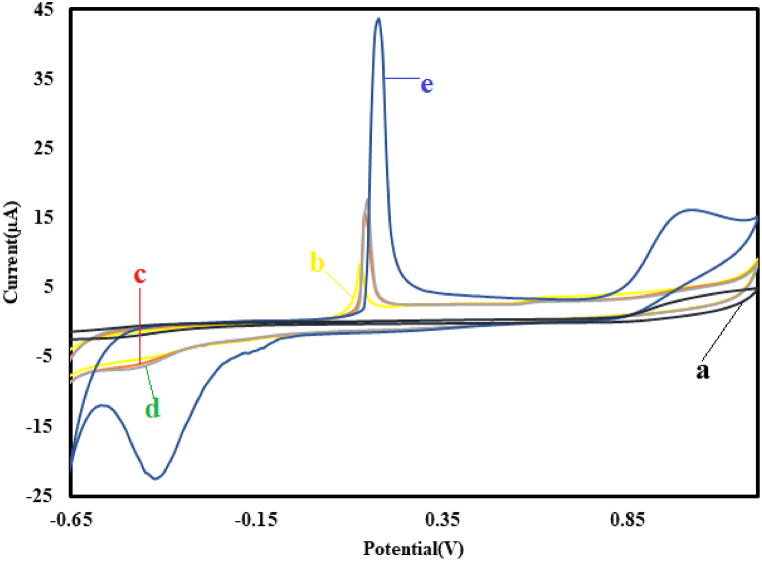
Analysis of nitrite on the surface of unmodified and modified electrodes: (a) bare GC electrodes in the presence of NO2-, (b) Cu@Ag-Nf/PA/GCE in the absence of nitrite, (c) PA/GCE and Cu@Ag-Nf/GCE, (d) Cu@Ag-Nf/PA/GCE, and (e) in the presence of nitrite.

Thus, the Cu@Ag-Nf/PA nanocomposite is an excellent choice for electrochemical nitrite detection, particularly at various applied potential ranges.

One potential mechanism could be suggested as follows [[47], [48], [49], [50], [51]]:

(1) Ag → Ag^+^ + e

(2) Ag^+^ + HNO_2_ → NO_2_ + H^+^ + Ag (OX1)

(3) Ag^+^ + NO_2_ + H_2_O → NO_3_^−^ + 2H^+^ + Ag

(4) Cu → Cu (I) + e

(5) Cu (I) + HNO_2_ → NO_2_ + H^+^ + Cu (OX2)

(6) Cu (I) + NO_2_ + H_2_O → NO_3_^−^ + 2H^+^ + Cu

(7) NO_3_-+ 9H^+^ + 8e → NH_3_ +3 H_2_O (Red)

Investigating the effect of pH is one of the key parameters to achieve the optimal response in electrocatalytic reactions. In this work, the behavior of the Cu@Ag-Nf/PA/GCE was studied at the pH rang of 2–4 and in the presence of nitrite analyte.

Above pH 4, only the electrocatalytic oxidation peaks (OX1 and OX2) are observed and the reduction peak (Red) is not active. With a decrease in pH, the peak oxidation decreases slightly and the peak reduction increases. This is probably due to the reduction of HNO_2_ to form NO (Eq. (8)) and then further reduction to N_2_O (Eqs. (9), (10), (11)) at lower pH [45,48].

(8) HNO_2_ + H^+^ + e^−^ → NO + H_2_O

(9) M (Cu, Ag) + HNO_2_ + H^+^ → NO + H_2_O + M (Cu^+^, Ag^+^)

(10) M (Cu, Ag) + NO + H^+^ → HNO + M (Cu^+^, Ag^+^)

(11) HNO + HNO → N_2_O + H_2_O

**Fig. 7:**
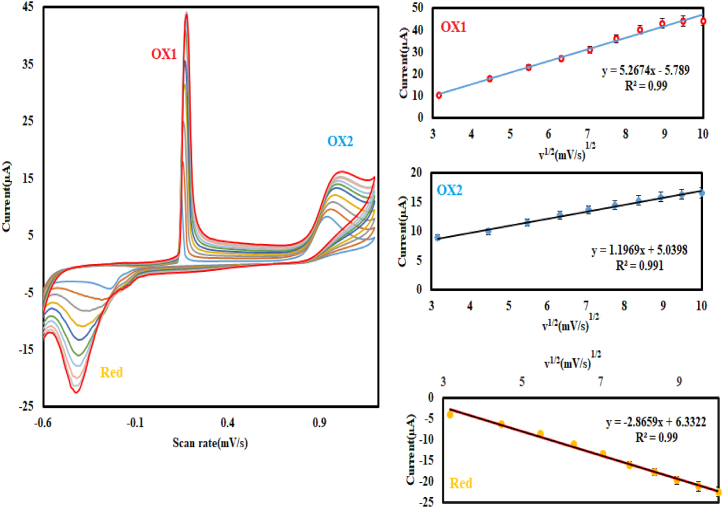
Cyclic voltammograms at different potential scan rates (10-100 mVs^−1^) show the response of Cu@Ag-Nf/PA/GCE in a phosphate buffer solution with 300 μM nitrite (pH = 2).

As a result, pH 2 was chosen as the optimal pH for future applications. The impact of varying scan rates (10-100 mVs^−1^) on the response of Cu@Ag-Nf/PA/GCE in a phosphate buffer solution containing 300 μM nitrite (pH = 2) was examined. The cathodic (Red) and anodic (OX1 and OX2) peak currents plotted against the square root of the scan rate exhibited linearity, suggesting diffusion control, as expected for a catalytic reaction (Fig. 7).

**Fig. 8:**
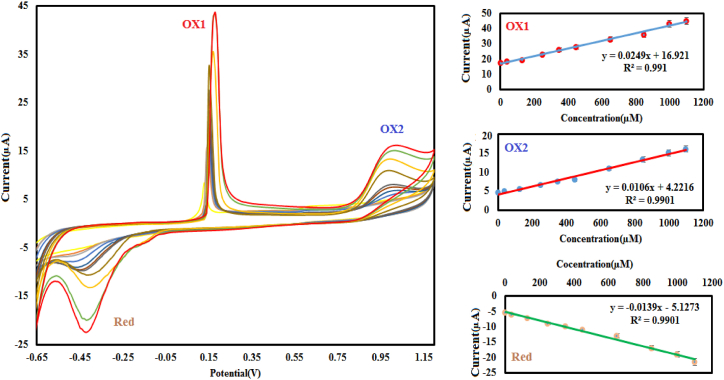
Cyclic voltammograms of Cu@Ag-Nf/PA/GCE in buffer solution at pH 2 with a scan rate of 20 mVs^−1^ and increasing nitrite concentration.

In Fig. 8, the performance of the modified electrode (Cu@Ag-Nf/PA/GCE) is assessed for different concentrations of nitrite ions. This electrode exhibited a strong response and high sensitivity to nitrite, showing three peaks simultaneously: two anodic peaks (OX1 and OX2) and one cathodic peak (Red) at +0.17, +0.98, and −0.42V, respectively. For the three peaks, the anodic and cathodic peak currents increased linearly with the nitrite concentration. Plots of the peak currents versus nitrite concentrations revealed the following results using the cycling voltammetry technique.

For OX1 peak: Equation: I (μA) = 0.0249[Nitrite] μAμM^−1^ + 16.921 μA, Correlation (R^2^ = 0.991), Linear range 2–1000 μM, Sensitivity: 0.0249 μAμM^−1^ and Detection of limit: 0.6 μM (signal/noise = 3)

For OX2 peak: Equation: I (μA) = 0.0106[Nitrite] μAμM^−1^ + 4.2216 μA, Correlation (R^2^ = 0.9901), Linear range 40–1100 μM, Sensitivity: 0.0106 μAμM^−1^ and Detection of limit: 9.8 μM (signal/noise = 3)

For Red peak: Equation: I (μA) = −0.0139[Nitrite] μAμM^−1^ - 5.1233 μA, Correlation (R^2^ = 0.9901), Linear range 2–1100 μM, Sensitivity: 0.0139 μAμM^−1^ and Detection of limit: 0.8 μM (signal/noise = 3)

**Fig. 9:**
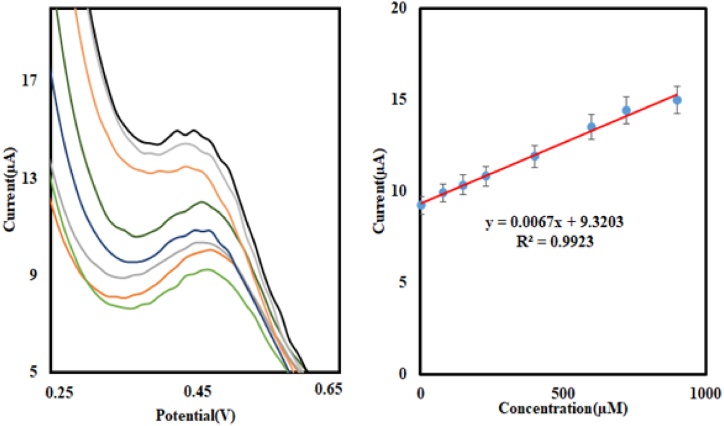
Differerntial puls voltammetry of Cu@Ag-Nf/PA/GCE in buffer solution at pH 2 with increasing nitrite concentrations.

The DPV technique was used to achieve a lower detection limit. Fig. 9 shows the linear relationship between the increasing anodic current and increasing nitrite concentration at pH 2. The corresponding results are as follows:

For DPV technique: Equation: I (μA) = 0.0067[Nitrite] μAμM^−1^ + 9.7203 μA, Correlation (R^2^ = 0.9901), Linear range 0.2–900 μM, Sensitivity: 0.0067 μAμM^−1^ with a Detection limit (signal/noise = 3) of 0.089 μM.

### 3.3 Repeatability and stability

A nitrite concentration of 200 μM was chosen to assess the consistency of voltammetric measurements of the Cu@Ag-Nf/PA/GC electrode at pH 2. The relative standard deviations (RSD) for five consecutive assays of OX1, OX2, and Red peaks were 6 %, 7 %, and 3 %, respectively. The long-term storage stability of the modified electrode was assessed over a 35-day period at room temperature. After 35 d, the proposed sensor maintained approximately 91 %, 87 %, and 93 % of its original response to the OX1, OX2, and Red peaks, respectively. These findings demonstrate the strong repeatability and stability of the Cu@Ag-Nf/PA/GC electrode for analyte analyses.

### 3.4 Comparison of the performance metrics of the suggested sensor with those of prior electrochemical techniques

Combining the benefits of core-shell nanoparticles and conductive polymers, the resulting Cu@Ag/Nf/PA exhibited a low detection limit and excellent electrocatalytic activity for nitrite ions. The proposed method was compared with other nitrite determination techniques. The results are presented in Table 1 [[52], [53], [54], [55], [56], [57], [58]]. It is evident that certain parameters in this study are similar to or better than those of other techniques.

**Table 1:** Analytical parameters of several modified electrodes for determination of nitrite.

Electrode	Method	LOD(μM)	Linear range(μM)	Sensivity	Ref
Fe_3_O_4_-NPs/GCE	AMP	5	500–7500	349.24 μA.mM^−^^1^cm^−2^	[52]
Iron (III)TMPP/CuTP/GCE	DPV	0.1	0.5–7.5	20.0 μA, 1 μmol-1	[53]
Chitosan/g-C_3_N_4_/GCE)	DPV	3	40–2000	0.12103 μA/μM	[54]
Au/Co_3_O_4_/GCE	SWV	0.11	1–4000	1.57 μA *μ*M-1 cm-2	[55]
gold microneedle	AMP	0.001	0.02–5.8	52.529 μA μM-1 cm-2	[56]
Na doped g-C_3_N_4_	CV	0.17	720–8150	426 μA.mM-1 cm- 2	[57]
Co_3_O_4_/GCE	Amp	0.22	6.6–3000	0.600 μA mM-1 cm-2	[58]
Cu@Ag-Nf/PA/GCE (OX1)	CV	0.6	40–1100	0.0249 μAμM−1	This work
Cu@Ag-Nf/PA/GCE (OX2)	CV	9.8	40–1100	0.0106 μAμM−1	This work
Cu@Ag-Nf/PA/GCE (Red)	CV	0.8	2–1100	0.0139 μAμM−1	This work
Cu@Ag-Nf/PA/GCE	DPV	0.089	900–0.2	0.0067 μAμM−1	This work

### *3.5* *Application of* Cu@Ag-Nf/PA/GC *for determination of nitrite*

As shown in Table 2, the Cu@Ag-Nf/PA/GC electrode was investigated for nitrite detection in two water samples using a recovery approach. The results showed acceptable data ranging from 99.1 to 101.36 %.

**Table 2:** Determination of nitrite in water samples.

Sample	Added (μM)	Found (μM)	Recovery (%)
River water	300	302.9	100.9
	600	604.7	100.78
Tap water	300	304.1	101.36
	600	595	99.1

### 3.6 Interference effects

To evaluate the selectivity of the Cu@Ag-Nf/PA/GC electrode, we investigated the effect of different potential interfering compounds in the water samples on the detection of 400 μM nitrite. The detection and determination of nitrite were not affected even when the concentrations of K^+^, F^−^, I^−^, IO_3_^−^, Br^−^, Cl^−^, Na^+^, Mg^2+^, Ca^2+^, Pb^2+^, SO_4_^2−^and CO_3_^2−^ were increased by 50 times.

## 4 Conclusion

A simple method was used to synthesize the Cu@Ag core shells. A novel electrochemical sensor was fabricated by immobilizing a Cu@Ag core–shell and conductive polymer on a glassy carbon electrode surface, which was used for the electroreduction and electro-oxidation of NO_2_^−^ for nitrite determination. We anticipate that the assembly of Cu@Ag-Nf/PA will result in the creation of a novel hybrid nanocomposite that exhibits enhanced properties for nitrite detection in water samples. It is also recommended that this sensor and the methods used to create it be applied in environmental monitoring, wastewater analysis and biomedical.

## CRediT authorship contribution statement

**Afshin Maleki:** Funding acquisition, Formal analysis, Conceptualization. **Nader Amini:** Writing – review & editing, Writing – original draft, Visualization, Project administration, Funding acquisition, Formal analysis, Data curation, Conceptualization. **Reza Rezaee:** Resources, Methodology. **Mahdi Safari:** Data curation. **Nader Marzban:** Funding acquisition. **Mehran seifi:** Conceptualization.

## Declaration of competing interest

The authors declare that they have no known competing financial interests or personal relationships that could have appeared to influence the work reported in this paper.
